# Varying Land-Use Has an Influence on Wattled and Grey Crowned Cranes’ Abundance and Distribution in Driefontein Grasslands Important Bird Area, Zimbabwe

**DOI:** 10.1371/journal.pone.0166209

**Published:** 2016-11-22

**Authors:** Togarasei Fakarayi, Clayton Mashapa, Edson Gandiwa, Shakkie Kativu

**Affiliations:** 1 Tropical Resource Ecology Programme, University of Zimbabwe, P. O. Box MP 167, Mount Pleasant, Harare, Zimbabwe; 2 BirdLife Zimbabwe, P. O. Box RVL 100, Runiville, Harare, Zimbabwe; 3 School of Wildlife, Ecology and Conservation, Chinhoyi University of Technology, Private Bag 7724, Chinhoyi, Zimbabwe; Pacific Northwest National Laboratory, UNITED STATES

## Abstract

Three species of cranes are distributed widely throughout southern Africa, but little is known about how they respond to the changes in land-use that have occurred in this region. This study assessed habitat preference of the two crane species across land-use categories of the self contained small scale commercial farms of 30 to 40 ha per household (A1), large scale commercial agriculture farms of > 50 ha per household (A2) and Old Resettlement, farms of < 5 ha per household with communal grazing land in Driefontein Grasslands Important Bird Area (IBA), Zimbabwe. The study further explored how selected explanatory (environmental) habitat variables influence crane species abundance. Crane bird counts and data on influencing environmental variables were collected between June and August 2012. Our results show that varying land-use categories had an influence on the abundance and distribution of the Wattled Crane (*Bugeranus carunculatus*) and the Grey Crowned Crane (*Belearica regulorum*) across Driefontein Grasslands IBA. The Wattled Crane was widely distributed in the relatively undisturbed A2 farms while the Grey Crowned Crane was associated with the more disturbed land of A1 farms, Old Resettlement and its communal grazing land. *Cyperus esculentus* and percent (%) bare ground were strong environmental variables best explaining the observed patterns in Wattled Crane abundance across land-use categories. The pattern in Grey Crowned Crane abundance was best explained by soil penetrability, moisture and grass height variables. A holistic sustainable land-use management that takes into account conservation of essential habitats in Driefontein Grasslands IBA is desirable for crane populations and other wetland dependent species that include water birds.

## Introduction

Cranes inhabit open habitats and are among the world’s most threatened bird families [1,2]. Southern Africa is home to three species of cranes, namely the Blue Crane (*Anthropoides paradiseus*), Grey Crowned Crane (*Balearica regulorum*) and Wattled Crane (*Bugeranus carunculatus*) [1,3,4]. Globally, Wattled Crane and Grey Crowned Crane are listed as vulnerable and endangered species, respectively, under the International Union of Conservation for Nature (IUCN) Red Data List [5,6]. Historically, Wattled Crane and Grey Crowned Crane species were widespread in Zimbabwe [2,7,8]. Currently, the distribution of cranes in the country is largely restricted to wet grasslands of the central plateau area of Zimbabwe, known as the Driefontein Grasslands [2]. This area is one of Zimbabwe’s 20 Important Bird Areas (IBAs), sites that are “hot spots” for bird species diversity [2,5,8,9]. The Driefontein Grasslands IBA area has experienced marked land-use changes since the year 2000 following the country’s resettlement programme [2,5,10]. Before the year 2000, the area was divided into large commercial livestock ranches, except one area where people were resettled in 1984, defined as the Old Resettlement in Zimbabwe [2].

Over the years, cranes were reported to have integrated well with livestock ranching, due to availability of protected natural wet grassland habitats [11–13]. Land-use has changed from large scale commercial cattle (*Bos taurus*) ranching to communal and subsistence mixed farming with relatively less preservation of land and vegetation as people tend to compete for available common forest resource reminiscent of the “tragedy of the commons” [2,14]. Three models of resettlement were adopted in Zimbabwe, firstly, the Old Resettlement of the 1980s, the villagized communal agriculture farms of < 5 ha per household with communal grazing land, lately, the self contained small scale commercial farms of 30 to 40 ha per household (A1) and large scale commercial agriculture farms of > 50 ha per household (A2) adopted in the 2000s. According to Fakarayi [2], the Zimbabwe’s land resettlement programme recorded land-use changes in Driefontein Grasslands IBA which constitute 20,000 ha. The proportion of land under wetlands cover was 17.5% in 1995 and decreased to 9.7% in 2010. The greatest reduction of wetlands and grassland cover classes occurred between 2005 and 2010, when 553 ha (22.2%) and 2,350 ha (20.3%) were lost respectively, whereas the land under cultivation increased from 89 ha in 1995 to 4,244 ha by 2010 in Driefontein IBA [2].

The new farming system of A1 in particular is characterised by transformation of some wet grasslands to cultivated lands [2]. The local farmers, who use wet grasslands for crop production and livestock grazing, shared this resource with bird species, e.g., cranes. However, this is perceived to affect cranes habitat use [10,12]. Rapid switch in land ownerships is associated with significant shifts in land-uses which results in habitat loss, destruction of traditional breeding sites, displacements of some species, reduced home ranges, deterioration of foraging sites and conflicts between human and birds on cultivated wetlands. This negatively affects species abundance, richness and diversity in an IBA due to restricted and disturbed habitats.

A scientific review on the status of cranes revealed that land-use change and management regime that have taken place in Driefontein Grasslands IBA were associated with general population decline of the crane species in the area since 2000 [8]. Land-use change due to human activities was reported as one of the factors that influenced the transformation of cranes’ habitat [1,15,16]. Elsewhere, it was recorded that human population pressures and associated land-use changes were major challenges hindering conservation efforts of cranes in developing countries where they occur [17]. As human population continues to increase, more land will likely be opened up for settlement and cultivation in the medium to long-term. Increased human activities in Driefontein Grasslands IBA could contribute to deterioration of the remaining natural crane habitat. Elsewhere in the southern African region, land-use change was cited as one of the major threats to cranes and other bird species [15,18].

There is a gap in knowledge on the responses of cranes to habitat modification in a changing land-use environment. In particular, little is known about the crane habitats, and the associated habitat features that are linked to crane abundance in a mosaic landscape. The impact of land-use change on the abundance and distribution of cranes, therefore, needs to be ascertained. The Driefontein Grasslands IBA supports a significant population of both Wattled Crane and Grey Crowned Crane in Zimbabwe. Besides being an indicator species for status of wetlands in the area, cranes are key species in this IBA. The area, therefore, is one of the priority sites for conservation if local extirpation of the crane species is to be averted. The study objectives were two-fold: (i) to determine habitat preference of the two crane species across land-use categories, and (ii) to establish how selected explanatory (environmental) habitat variables (i.e., soil penetrability, vegetation structure composition, foraged *Cyperus esculentus* and grass cover) influence crane species abundance. Understanding the distribution of cranes in response to habitat changes is crucial for development of conservation management strategies for the target species in Driefontein Grasslands IBA.

## Materials and Methods

### Study area

**Fig 1:**
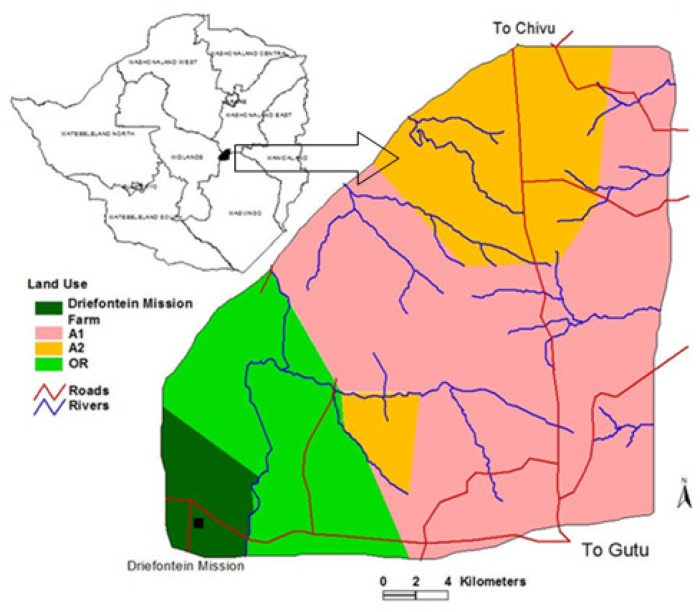
Location of Driefontein Grasslands Important Bird Area, Zimbabwe. Where A1 = the self contained small scale commercial farms of 30 to 40 ha per household, A2 = large scale commercial agriculture farms of > 50 ha per household and OR = Old Resettlement, villagized agriculture farms of < 5 ha per household with communal grazing land. Source: [2].

Driefontein Grasslands IBA is located outside the protected area system of Zimbabwe, and about 30% part of it is privately owned (e.g., Driefontein Mission farm and other few A2 farms), whereas, the larger part of it about 70% is under communal land comprised of A1 and Old settlement (Fig 1). Study permission was sought and granted by the local authority (Gutu Rural District Council), private (including Driefontein Mission) and communal land owners of the Driefontein Grassland IBA. Driefontein Grasslands IBA (20,000 ha) is located on Zimbabwe’s central plateau (19° 23' S; 30° 47' E) and is characterized by extensive expanses of open grasslands, wetlands and cultivated land [2]. The study area is characterised by a semi-arid climate with a mean annual rainfall of about 650 mm and annual temperatures range from 12°C to 32°C [9]. There are a few other rivers such as Nyororo and Shashe. The soils are sandy and fast-draining, except where water runs into shallow clay-lined depressions, called vleis, which support dense reed beds [8,9]. Most of the area is not well-suited to crop production [12], and was divided into large commercial cattle ranches until the onset of Zimbabwe land redistribution program in the year 2000, where land-use categories like communal land-use for settlement and agriculture were introduced.

### Study strata and sampling procedure

The study area was stratified into the three defined land-use categories of A1, A2 and Old Resettlement areas of Driefontein Grassland IBA [2]. Bird census points were randomly assigned to the three defined land-use categories across the study area following Fakarayi *et al*. [2]. The computer generated cranes census points were verified for accessibility before commencing cranes counting using a hand held Garmin Geographic Positioning System (GPS) 60 receiver unit (Garmin Ltd, Olathe, Kansas, USA). Distance from one station of bird census count to another point was about 500 m. This spacing allowed for greater coverage of the study area and also moderate to high sampling intensity. Twenty two bird census points were randomly selected on the A1 land-use category study stratum, 17 from the A2 land-use category and six from the Old Resettlement study stratum, this ratio of bird census points was in relation to the land size of each of the three study stratum. At each bird census point, two one hour long observations were made (06:00–07:00 and 16:00–17:00) and crane species and their numbers recorded separately for each species. The cranes on each census point were surveyed and all individual seen or heard once were recorded (S1 File).

Ten ground surveys of cranes on each bird census point were carried between 15 June and 18 August 2012 to determine the distribution of cranes across the defined land-use categories of the study area. Replication of sampling on repeated bird census points was done to increase reliability and applicability of the results as recommended by Bibby *et al*. [19]. Locations of individuals, pairs or flocks of cranes were determined by searching all census points and one hour long continuous focal observations were also carried out at dawn (06:00–07:00) and dusk (16:00–17:00) throughout the survey period. All cranes sighted were identified to species level and counted. Habitat attributes were measured to explain bird distribution and habitat preferences in the study area. A plot-based method was used to measure habitat attributes, a method adopted from Javed and Kaul [20]. Square quadrat plots of 1 m^2^ were established on each crane census point across the three study strata. A plot size of 1 m^2^ was adopted in this study as it was the recommended size for measuring grasses and herbs [20].

In each quadrat, grass height was measured using a 2 m tape measure. The plant density was calculated through physical total counting of all grasses in a quadrat and expressed in relation to the quadrat area, whereas percent grass cover and/or bare ground were visually estimated in the quadrat [19]. Ground condition was characterized as wet or dry, soil penetrability was measured using a graduated point stick that was dropped at the centre of each quadrat from a height of 1.5 m above ground level and the depth to which it penetrated was recorded [19]. The number of perennial sedge per quadrat was determined through direct total counts. Sizes of sampling units of the same habitats were combined in each land-use category using Quantum Geographical Information System (GIS), ArcView GIS and Google mapping [21–23] and density of birds was then calculated per land-use category. Density was calculated as total number of birds recorded from crane census points of the same land-use category expressed as a proportion of total habitat size of each land-use category.

### Data Analysis

#### Habitat preference by cranes in Driefontein Grassland IBA

Habitat preference by crane species was evaluated using Habitat Preference Indices (HPIs). The HPI for each crane species was calculated and analyzed per habitat type in each land-use category following Javed and Kaul [20]. These HPIs were calculated as percentage habitat use for each crane species divided by percentage habitat availability. Percentage habitat use for each crane species was determined as the number of crane species recorded in a specific habitat type over the study period, divided by the total number of crane of the same species tallied per census per land-use model multiplied by 100. The percentage habitat availability was established as the number of occurrences of a habitat type in a land-use category divided by the total number habitats sampled in that land-use category multiplied by 100.

Furthermore, a two-way analysis of variance (ANOVA) was performed in GenStat for Windows 14^th^ Edition (VSN International, Hemel Hempstead, UK) to compare crane species abundances across the three land-use categories. The two factors were denoted as land-use category (with three levels) and crane species (two levels) or habitat type (two levels) and crane species (two levels). This was done upon testing the count data for normality using Q-Q plots in Statistical Package for Social Sciences (SPSS) Version 16.0 for Windows (IBM SPSS Inc, Chicago, USA). Non-normal data were log transformed prior to analysis to conform to normality distribution. A *post-hoc* test using Fisher’s Least Significance Difference (LSD) was performed for the variable land-use to determine significant differences in crane abundances between land-use category sites. A Welch two-sample *t*-test (an unequal variance *t*-test) was used to compare mean abundances of cranes between cultivated and wetland habitats in each land-use category.

#### Crane–environment relationships

A Redundancy Analysis (RDA) with unrestricted Monte Carlo permutations was performed using CANOCO for Windows (version 4) [24] to investigate the relationship between the two crane species and the measured habitat explanatory variables that may influence their distribution and abundance. Prior to RDA, a Detrended Correspondence Analysis (DCA), an indirect gradient method [24] was used to determine suitability of RDA which was met given that the length of the gradient was less than 4.

## Results

### Habitat preference by cranes in Driefontein Grassland IBA

**Fig 2:**
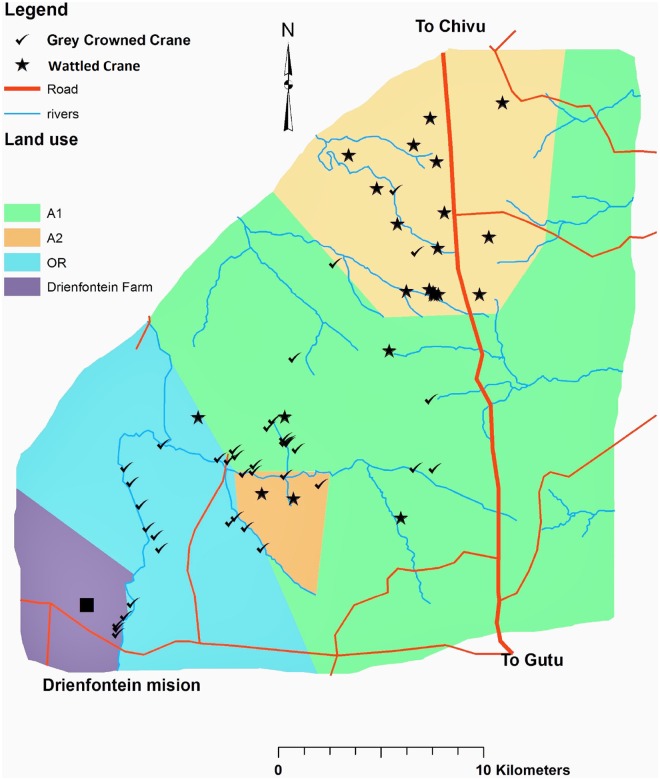
Locations of crane sightings in the Driefontein Grasslands IBA, Zimbabwe. Where A1 = the self contained small scale commercial farms of 30 to 40 ha per household, A2 = large scale commercial agriculture farms of > 50 ha per household and OR = Old Resettlement, villagized agriculture farms of < 5 ha per household with communal grazing land. Source: [2].

Cultivated land under the A2 land-use category was the preferred foraging habitat for the Wattled Cranes (Table 1). The A1 and Old Resettlement categories supported the majority of the Grey Crowned Crane (Fig 2). In both A1 and A2 resettlement models, the cultivated habitat was more preferred than the wetland habitat, whereas in Old Resettlement, the wetland habitat was more preferred than the cultivated habitat. Habitat preference of the Wattled Crane was not significantly influenced by the land-use categories (χ^2^ = 0.10, df = 2, *p* = 0.95). Wetland habitats in land-use categories Old Resettlement and A2, and cultivated habitats in A1, had higher than expected proportions of foraging by the Grey Crowned Crane. No association between habitat preference of Grey Crowned Crane and land-use models was recorded (χ^2^ = 0.33, df = 2, *p* = 0.848).

**Table 1:** Habitat Preference Indices (HPIs) for the Wattled Crane and Grey Crowned Crane in Driefontein Grasslands IBA.

Land-use Category	Habitat	HPI	
		WC	GCC
A1	Cultivated field	1.05	1.15
	Wetland	0.93	0.80
A2	Cultivated field	1.22	0.72
	Wetland	0.86	1.19
Old Resettlement	Cultivated field	0.83	1.77
	Wetland	1.08	1.67

Notes: WC = Wattled Cranes and GCC = Grey Crowned Cranes. A HPI of 1 suggests cranes were using a specific habitat type in proportion to its availability, a HPI >1 suggest use higher than expected, whereas a value <1 suggests avoidance [20]. A1 = land-use category under the self contained small scale commercial farms of 30 to 40 ha per household, A2 = land-use category under large scale commercial agriculture farms of > 50 ha per household and Old Resettlement = land-use category under villagized agriculture farms of < 5 ha per household with communal grazing land.

**Fig 3:**
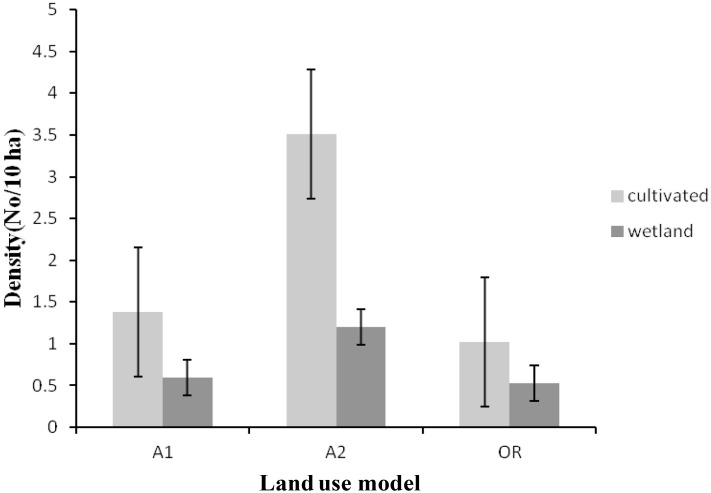
Densities of Wattled Crane for each land-use category in 2012. Where A1 = the self contained small scale commercial farms of 30 to 40 ha per household, A2 = large scale commercial agriculture farms of > 50 ha per household and OR = Old Resettlement, villagized agriculture farms of < 5 ha per household with communal grazing land. Error bars indicate level of error margin (±0.05) or uncertainty of population densities recorded in this study.

**Fig 4:**
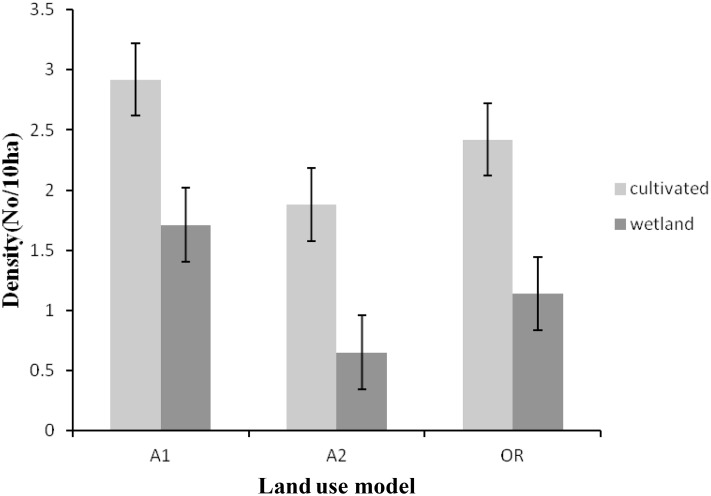
Densities of the Grey Crowned Crane for each land-use category in 2012. Where A1 = the self contained small scale commercial farms of 30 to 40 ha per household, A2 = large scale commercial agriculture farms of > 50 ha per household and OR = Old Resettlement, villagized agriculture farms of < 5 ha per household with communal grazing land. Error bars indicate level of error margin (±0.05) or uncertainty of population densities recorded in this study.

The density of Wattled Crane was highest in the cultivated habitat, especially in the land-use category A2, for all the three land-use categories (Fig 3). Similarly, the density of Grey Crowned Crane was high in cultivated habitat, in particular in the land-use category A1, for all the three land-use categories (Fig 4).

The A2 land-use category had the highest number of Wattled Cranes. In the A1 and Old Resettlement land-use categories, 50% of the Wattled Crane counts in both wetland and cultivated habitats were below 10 birds. Similarly, for A2 land-use category, 50% of counts in wetland habitats had less than 10 birds. Significant difference in Wattled Crane abundance was recorded across three land-use categories (*F*_2,164_ = 6.13, *p* = 0.003); Fisher’s LSD *post hoc* results Wattled Crane abundances between land-use A1 and A2 (*p* = 0.029), A2 and Old Resettlement (*p* = 0.001), and A1 and Old Resettlement (*p* = 0.123) land-use categories. Habitat type had no significant (*F*_1,164_ = 3.45, *p* = 0.065) influence on Wattled Crane abundances, but an interaction of land-use model and habitat type had a significant (*F*_2,164_ = 3.91, *p* = 0.022) influence on Wattled Crane abundances. The Welch two-sample t-test for independent samples showed significant (*t* = 4.03, df = 68, *p* = 0.036) difference of Wattled Crane abundances between cultivated and wetland habitats for A2 land-use model. In A1 (*t* = 0.56, df = 68, *p* = 0.329) and Old Resettlement (*t* = -0.63, df = 28, *p* = 0.792) models the *t*-test for independent samples revealed no significant difference in crane abundances between the two habitats.

The cultivated habitat had a greater variation in abundance of Grey Crowned Crane than the wetland habitat. About 50% of the Grey Crowned Crane counts in both cultivated and wetland was below 10 birds in land-use categories A2 and Old Resettlement. A two-way analysis of variance revealed a statistically significant (*F*_2,164_ = 17.33, *p* = 0.001) difference in Grey Crowned Crane abundance across the three land-use categories. A *post hoc* test (Fisher’s Least Significant Differences of means) revealed significant differences in abundances between A1 and A2 (*p* = 0.0001), A2 and Old Resettlement (*p* = 0.002), but no significant difference between land-uses A1 and Old Resettlement (*p* = 0.152) land-use categories. The Welch two-sample *t*-test for independent samples revealed no significant difference in Grey Crowned Crane abundance between cultivated and wetland habitats in all three land-use categories of A1 (*t* = 1.46, df = 28, *p* = 0.056), A2 (*t* = -0.48, df = 28, *p* = 0.787) and Old resettlement (*t* = -0.48, df = 28, *p* = 0.787) models.

### Crane–environment relationships

Only two of the measured variables, i.e., gradient of *Cyperus esculentus* and soil penetrability had significant influence on abundances of the two crane species (Table 2). The RDA results showed that the first two axes accounted for 77.6% of species data and 100% of species-environmental variation of the crane species-environment relationship.

**Table 2:** RDA unrestricted permutation Monte Carlo showing significant influence of measured habitat variables on the two crane species.

Habitat variable	*F* ratio	*p* value
*Cyperus esculentus*	8.65	0.004*
Soil penetrability	6.03	0.026*
Soil moisture	2.00	0.158
Plant species richness	4.69	0.050
% grass cover	0.75	0.380
Average grass height	0.68	0.442
Plant density	0.25	0.706
% bare ground	0.25	0.642
Distance from nearest settlement	0.12	0.802

The symbol * represents statistical significance, *p* < 0.05

The first axis defined a gradient of *C*. *esculentus* in open habitat with high soil penetrability. The second axis defined soil moisture in association with plant species richness and average grass height gradient. The first and second axes showed very strong correlations between crane species and the environment (Table 3). The third and fourth axes showed no correlation between crane species and the environment. These axes defined the distance between foraging habitat and human settlement.

**Table 3:** Eigenvalues, correlations and cumulative percentage variance from the RDA.

Variable	Axes			
	1	2	3	4
Eigenvalues	0.50	0.28	0.22	0.01
Species-environment correlations	0.88	0.89	0.00	0.00
Cumulative percentage variance of species data	50.00	77.60	99.40	100.00
Cumulative percentage of species-environment relationship	64.40	100.00	0.00	0.00

**Fig 5:**
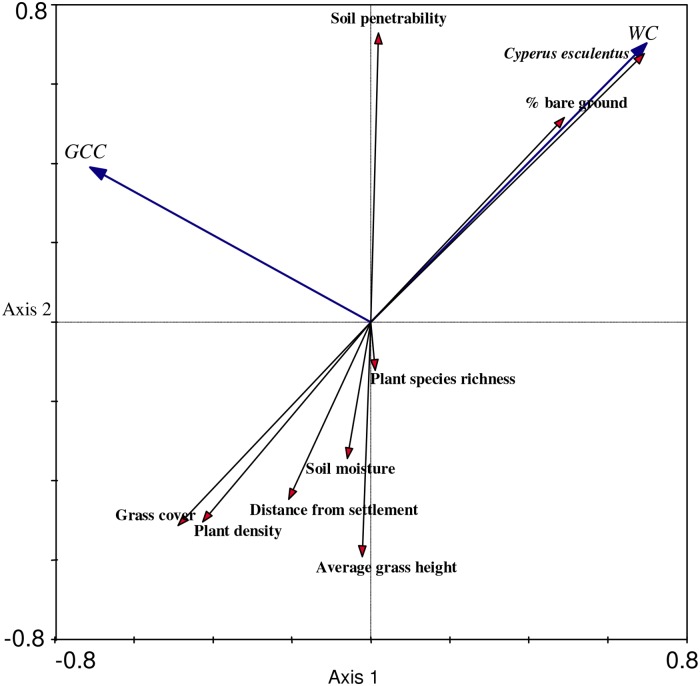
Redundancy Analyses diagram displaying the first two ordination axes on the influence of environmental habitat variables on crane species abundance. Note: WC = Wattled Crane and GCC = Grey Crowned Crane.

Wattled Crane was positively correlated with *C*. *esculentus* and % bare ground (Fig 5). This showed a high Wattled Crane abundance in open habitats with high densities of the perennial sedge plant (*C*. *esculentus*). Soil penetrability had also a positive influence on the Wattled Crane abundance. High abundance of Grey Crowned Crane was linked to high values of soil penetrability, low grass cover, and plant density. *C*. *esculentus* and % bare ground also positively influenced high abundance of Grey Crowned crane, but to a lesser extent. The variables soil penetrability, *C*. *esculentus* and % bare ground were negatively correlated with the rest of the variables.

## Discussion

Cultivated habitat in the Old Resettlement land-use category had higher than expected proportions of foraging Grey Crowned Crane (i.e., HPI = 1.77). The high preference of cultivated habitat could be explained by easily accessible food (*C*. *esculentus*) as the habitat had high soil penetrability. This corroborates with Muheebwa [25] who related cranes to food availability in crop fields. This relationship, however, was more evident to the Grey Crowned Crane. In land-use category A2, cultivated habitat preference by the Wattled Crane could be directly related to the gradient of *C*. *esculentus* in combination with openness of the habitat (Fig 5). Another possible explanation for this could be that some of the cultivated habitats were newly converted wetlands as reported by Fakarayi *et al*. [2]. These newly cultivated wetlands possess similar characteristics as wetlands, and therefore, are attractive to Wattled Cranes; hence the reason for the recorded high number of Wattled Cranes in cultivated lands. This explains effects of habitat transformation that has taken place as a result of land-use in Driefontein Grasslands IBA. However, it remains unclear how sustainable this would be since Wattled Cranes are less resilient to environmental changes. The two crane species had different habitat preferences in land-use category A2. According to Muheebwa [25], farming practices frequently influence the number of cranes using a particular field. This could explain recorded different levels of habitat preferences by both species of cranes in cultivated habitats across the three land-use categories.

Our study has shown that the Wattled Crane is largely distributed in the northern part of Driefontein Grasslands IBA within the section of A2 large scale farms (Fig 2). It is possible that the flocks of Wattled Cranes observed could have been splitting and occupying various habitats within the A2 land-use category, resulting in a wide distribution within this land-use. The Grey Crowned Cranes were mainly distributed within the south-western part of the Driefontein Grasslands IBA occupying habitats in A1 and Old Resettlement land-use categories. These findings are contrary to the findings of Couto and Couto [12] and Mundy *et al*. [10] who previously reported evenness on the distribution of crane species in Driefontein Grassland IBA. The present study highlighted that it is likely the two crane species have shifted from their historical sites and occupied new sites. This could be attributed to changes in land-use in the study area [2].

The significant differences in abundances of both Wattled Crane and Grey Crowned Crane, across the three land-use categories (Figs 3 and 4) could be attributed to nature of land-use categories in terms of land size per unit household and level of disturbances. The A2 land-use category is characterized by large farms with minimum land disturbances which could be the reason why it attracted the highest number of Wattled Cranes. The Wattled Crane is known to be more sensitive to land disturbances; therefore, it tends to move to less disturbed habitat [16]. Wattled Crane requires more space and its home range has been recorded to be around 16.6 km^2^, with 2.3% of this being the core breeding area [16]. Other authors [7,16] have reported that the Wattled Crane require a less disturbed habitat, and this could explain the very low abundance of the Wattled Crane in A1 and Old Resettlement categories, which were characterized by high levels of land disturbances as reported by Fakarayi *et al*. [2].

The lack of a significant difference in abundance of Grey Crowned Crane between land-use categories A1 and Old Resettlement could be due to similar land-use practices characterized by high human disturbances in these two land-use categories [2]. The Grey Crowned Crane was associated with densely human populated land-use category. This corroborates the findings of Meine and Archibald [15] that related Grey Crowned Cranes to landscape within high human population densities in KwaZulu-Natal, South Africa. Moreover, our findings are similar to those of Chirara [8] who reported a higher adaptive capacity of Grey Crowned Crane than the Wattled Crane across the same study area.

The selected explanatory variables in the present study proved to be good predictors as they explained 77.6% of species data across the study area (Table 3). It should be noted that the environmental variables considered in this present study, however, are by no means exhaustive although they explained the greatest variation in crane species abundance and distribution. Other variables or factors to consider in future studies include the scale of study, grazing pressure, and stochastic processes (e.g., fire). According to Chirara [8], fire is prevalent in the Driefontein Grasslands IBA especially during the dry season, and therefore could be influencing bird species distribution. In literature [4,9,11], both species of cranes are strongly related to wet ground, but in contrast, moisture was not among the strong variable explaining the variation in this present study (Table 2). The possible explanation for this disparity could be the time when this study was carried out (i.e., dry season) and that most crane sightings were in cultivated and ploughed crop fields that quickly lose out moisture due to loosened soils. This also explained high soil penetrability in a dry cultivated habitat. Positive correlation between both species of cranes and *C*. *esculentus* explains preference of this plant species as food by the two crane species in Driefontein Grasslands. Presence of this plant species *C*. *esculentus* in cultivated lands as indicated by our results could be another reason of high abundance of both species of cranes in cultivated lands.

## Conclusion

The varying land-use categories in Driefontein Grasslands IBA had an influence on the abundance and distribution of the Wattled Crane and the Grey Crowned Crane across the study area. Moreover, changes in land-use had an influence on habitat types. Habitat types were preferred differently by the two crane species as influenced by different land-uses. The Wattled Crane was widely distributed in relatively conserved large scale agricultural farms while the Grey Crowned Crane was associated with disturbed land of communal agricultural farms. The Grey Crowned Crane seem more resilient and adaptive to habitat changes than the Wattled Crane. Conservation of essential habitats is critical for crane populations and other biodiversity because humans frequently alter shallow marshes and wet grasslands [2] which are important crane nesting habitats, thus, land managers and planners need to better understand crane habitat preferences and whether habitat changes influence the success of cranes populations. Since the study was carried out in one season, there is need to investigate the crane abundance and distribution as well as the environmental factors in a different season for comparative purposes.

## Supporting Information

S1 File Crane sightings in Driefontein Grassland IBA, Zimbabwe. (XLS)

## References

[1] Beilfuss RD, Dodman T, Urban EK (2007) The Status of cranes in Africa in 2005. *Ostrich* 78(2), 175–184.

[2] Fakarayi T, Mashapa C, Gandiwa E, Kativu S (2015) Pattern of land-use and land cover changes in Driefontein Grassland Important Bird Area, Zimbabwe. *Tropical Conservation Science*, 8 (1), 274–283

[3] Harrison JA, Allan DG, Underhill LG, Herremans M, Tree AJ, Parker V (eds) (1997) The Atlas of Southern African Birds. BirdLife South Africa: Johannesburg, Vol 1, 316–317.

[4] Morrison KL (1998) Habitat utilization and the population ecology of cranes in the Dullstroom area of the Mpumalanga province. MSc Thesis. University of Pretoria: Pretoria.

[5] IUCN (2015a) IUCN Red List of Threatened Species. Version 2012.1. Accessed on 05 September 2015. www.iucnredlist.org

[6] IUCN (2015b) IUCN Red List of Threatened Species. Version 2013. <www.iucnredlist.org>. Downloaded on 17 Jamuary 2016.

[7] Hockey PAR, Dean WRJ, Ryan PG (eds) (2005) Roberts Birds of Southern Africa. Trustees of the John Voelcker Bird Book Fund: Cape Town.

[8] Chirara C (2011) The status of the Wattled Crane *Bugeranus carunculatus* in the Driefontein Grasslands of Zimbabwe. *Honeyguide* 57, 10–14.

[9] Childes SL, Mundy PJ (2001) Important Bird Areas in Africa and Associated Islands-Zimbabwe In Fishpool LDC and Evans MI (eds), Important Bird Areas in Africa and associated Islands: *Priority sites for conservation*. First edition Nature Bureau, United Kingdom pp 1025–1042.

[10] Mundy PJ, Maozeka F, Couto JT (2001) An update on the status of Wattled Cranes in Zimbabwe. *Honeyguide* 47(2), 129–134.

[11] Rockingham-Gill D (1996) Water, wetlands, and cranes in Lomagundi. In Beilfuss RD, Tarboton WR, Gichuki NN (eds) Proceedings of the African Crane and Wetland Training Workshop. Baraboo, Wisconsin: International Crane Foundation, 337–340.

[12] Couto JT, Couto F M (2000) Summary of a ground census for Wattled Cranes in 1996. *Honeyguide* 46(2), 123–132

[13] Maozeka F (2000) Update on cranes and an overview of the work of the Zimbabwe Crane Working Group (ZCWG) in Zimbabwe. Proceedings of the 12th South African Crane Working Group Workshop.

[14] Hardins G (1968) The tragedy of the commons. *Science* 162, 1243–1247. 10.1126/science.162.3859.1243 17756331

[15] Meine CD, Archibald GW (eds.) (1996) The Cranes, Status Survey and Conservation Action Plan. IUCN, Gland, Switzerland, and Cambridge, U.K., Northern Prairie Wildlife Research Center, 294 pp.

[16] McCann KI, Benn GA (2006) Land-use patterns within Wattled Crane (*Bugeranus carunculatus*) home range in KwaZulu-Natal, South Africa. *Ostrich*, 77(3&4), 186*–*194.

[17] Allan DG, Harrison JA, Navarro RA, van Wilgen BW, Thompson MW (1997) The impact of commercial afforestation on the bird populations in Mpumalanga Province, South Africa: insights from bird-atlas data. *Biological Conservation* 79, 173–185

[18] Bento CM, Beilfuss RD, Hockey PAR (2007) Distribution, structure and simulation modelling of the Wattled Crane population in the Marromeu Complex of the Zambezi Delta, Mozambique. *Ostrich* 78(2), 185–193.

[19] Bibby C, Jonnes B, Marsden S (1998) Expedition Field Techniques Bird Surveys. Expedition Advisory Centre, London.

[20] Javed S, Kaul R (2002) Field Methods for Bird Surveys. Bombay Natural History Society; Department of Wildlife Sciences, Aligarh Muslim University, Aligarh, and World Pheasant Association, South Asia Regional Office (SARO), New Delhi, India.

[21] Giri C, Jenkins C (2005) Land cover mapping of greater Mesoamerica using MODIS data. *Canadian Journal of Remote Sensing* 31, 274–282.

[22] Buchanan GM, Butchart SHM, Dutson G, Pilgrim JD, Steininger MK, Bishop D et al (2008) Using remote sensing to inform conservation status assessment: estimates of recent deforestation rates on New Britain and the impacts upon endemic birds. *Biological Conservation* 141, 56–66.

[23] Allnutt TF, Asner GP, Golden CD, Powell GVN (2013) Mapping recent deforestation and forest disturbance in northeastern Madagascar. *Tropical Conservation Science* 6,1–15.

[24] ter Braak, CJF, Šmilauer P (2002) CANOCO Reference manual and CanoDraw for Windows User’s guide: Software for Canonical Community Ordination (version 4.5), Microcomputer Power, Ithaca, New York.

[25] Muheebwa JM (2004) Assessing the status of the Grey Crowned Crane Balearica regulorum in Uganda. Unpublished MSc Thesis. Makerere University: Kampala.

